# Allergic diseases do not impair the cognitive development of children but do damage the mental health of their caregivers

**DOI:** 10.1038/s41598-020-70825-1

**Published:** 2020-08-17

**Authors:** Ho-Chang Kuo, Ling-Sai Chang, Zi-Yu Tsai, Liang-Jen Wang

**Affiliations:** 1 Department of Pediatrics and Kawasaki Disease Center, Kaohsiung Chang Gung Memorial Hospital, Kaohsiung, Taiwan; 2 Chang Gung University College of Medicine, Kaohsiung, 83301 Taiwan; 3 Department of Child and Adolescent Psychiatry, Kaohsiung Chang Gung Memorial Hospital and Chang Gung University College of Medicine, No.123, Ta-Pei Road, Kaohsiung, Taiwan

**Keywords:** Allergy, Asthma

## Abstract

This study aimed to investigate whether children with atopic diseases exhibited different neurodevelopment function from healthy controls and whether their caregivers had differential parental stress. In total, we recruited 109 patients with atopic diseases (mean age 6.8 years, 54.1% male) and 82 healthy children (mean age 6.3 years, 54.9% male). Based on the children’s age, they underwent developmental, cognitive evaluations and attention deficit/hyperactivity disorder (ADHD) symptoms. The parenting stress of children’s caregivers was evaluated using the Chinese Health Questionnaire (CHQ-12) and Family APGAR. Of the children with atopic diseases, 87.2%, 74.3%, 29.4%, and 8.3% of them had allergic rhinitis, asthma, atopic dermatitis, and urticaria, respectively. None of these conditions were associated with children’s cognitive profiles or ADHD symptoms. However, the caregivers of patients who had asthma suffered from higher CHQ-12 scores than those of patients without asthma. Furthermore, the number of atopic diseases had a dose–response effect on caregivers’ CHQ-12 scores. In conclusion, allergic diseases did not impair the cognitive development of children. However, caregivers of patients with asthma or multiple atopic diseases may suffer a greater mental health burden with regard to caring for their children. Such caregivers may require support to effectively fulfill their parenting roles.

## Introduction

Atopy refers to the genetic susceptibility to developing allergy-related diseases^1^. Atopic diseases, including allergic rhinitis^2^, asthma^3^, atopic eczema/dermatitis^4^, and urticaria^5^ are common in children and present many management challenges for their caregivers^6–8^. Allergies are immune responses and usually involve chronic inflammation^9^. However, whether inflammation and irritation increase the risk of neuropsychological consequences in children with atopic diseases is still unclear^10–12^. For example, allergic rhinitis is associated with significantly impaired mental health and impaired cognitive function^13^. Asthma is a chronic respiratory disease, but has no negative impact on patients’ intellectual quotient (IQ)^14^.

In addition, some studies have shown that the relationship between immune response and the central nervous system (CNS) may make children more susceptible to neuropsychological disorders, such as attention deficit hyperactivity disorder (ADHD)^15^. Various allergic diseases, such as allergic rhinitis, atopic dermatitis, or asthma, have been associated with ADHD^16–19^. Several epidemiological studies using large databases have suggested that ADHD is associated with atopic dermatitis in children^20,21^. However, another population study revealed that children with atopic dermatitis did not have a significantly increased prevalence of ADHD^22^.

In addition, caring for children with atopic diseases can be a time-consuming task that can lead to mental health burdens on the caregiver and cause a decline in his or her psychosocial function^23,24^. Deficient sleep with poor quality in the caregivers of children with chronic illnesses may have a significant impact on their health and well-being, as well as on their caregiving responsibilities^25^. However, it is unclear whether specific atopic diseases affect patients' cognitive development or impose pressure on caregivers. Moreover, atopic diseases are usually comorbid, and whether the number of atopic diseases showed a dose-related effect on patients’ neurocognitive outcomes and caregivers’ parental stress remains unclear.

We hypothesized that chronic atopic diseases may have detrimental effects on neurodevelopment, and caregivers of patients with atopic diseases may experience great mental health burden with regard to caring for their children. Therefore, we conducted a clinical survey to investigate whether children with atopic diseases and healthy controls exhibit different neurodevelopmental functions, and whether their caregivers have different parental stressors. We also investigated whether the number of atopic diseases showed a dose-related effect on the outcomes of the above mentioned children and parents.

## Results

The clinical cohort consisted of 109 patients with atopic diseases (mean age 6.8 years, 54.1% male) and 82 healthy children (mean age 6.3 years, 54.9% male) (Table 1), with no significant differences in age or gender between the atopic disease children and the controls. We observed no significant differences in the cognitive scores or SNAP-IV scores between the children with atopic diseases and the healthy controls. With regard to caregiver characteristics, no significant difference in age, sex, or relation to the patients was observed between the caregivers of patients with atopic diseases and those of the controls. Furthermore, the caregivers of the atopic disease children had higher CHQ scores than the control group, but we found no difference in family APGAR scores.

**Table 1:** Characteristics and development of children with allergic diseases and control subjects.

Variables	Allergic diseases (N = 109)		Controls (N = 82)		Statistics	P-value
	Mean or N	SD or %	Mean or N	SD or %		
**Characteristics of children**						
Age (years)	6.8	2.3	6.3	3.0	1.113	0.267
**Sex**					0.011	0.918
Female	50	45.9	37	45.1		
Male	59	54.1	45	54.9		
**Allergic diseases**						
Allergic rhinitis	95	87.2	–			
Asthma	81	74.3	–			
Atopic dermatitis	32	29.4	–			
Urticaria	9	8.3	–			
**Number of allergic diseases**						
1	23	21.1	–			
2	65	59.6	–			
≥ 3	21	19.3	–			
**Outcomes**						
Intelligence Quotient	108.9	13.7	108.0	16.5	0.423	0.673
SNAP-IV	24.6	10.7	22.7	14.2	0.928	0.355
**Characteristics of caregivers**						
Age (years)	38.8	4.9	38.0	4.9	1.146	0.253
**Sex**					0.063	0.802
Female	88	80.7	65	79.3		
Male	21	19.3	17	20.7		
**Education levels**					0.894	0.640
High school or lower	37	33.9	28	34.1		
College	56	51.4	38	46.3		
Master or above	16	14.7	16	19.5		
**Relation to the patients**					0.106	0.948
Mother	84	77.1	62	75.6		
Father	18	16.5	15	18.3		
Others	7	6.4	5	6.1		
**Mental Health**						
CHQ	3.1	2.0	2.1	2.4	3.069	0.003
Family APGAR	7.5	2.8	7.4	2.7	0.158	0.874

Data are expressed as mean ± SD or n (%).

Of the children with atopic diseases, 87.2%, 74.3%, 29.4%, and 8.3% of them suffered from allergic rhinitis, asthma, atopic dermatitis, and urticaria, respectively. Table 2 shows the effects of allergic diseases on children’s cognitive function and caregivers’ mental health. We found that asthma was positively correlated to caregivers’ CHQ scores (t = 3.069, p = 0.003). However, allergic rhinitis, asthma, atopic dermatitis, and urticaria did not exhibit an individual effect on children’s cognitive scores, SNAP-IV scores, or caregivers’ family APGAR scores. Furthermore, the caregivers’ education levels of a college’s degree or above were associated with higher cognitive scores, lower CHQ scores, and higher family APGAR scores.

**Table 2:** The effects of allergic diseases on children’s cognitive function and caregivers’ mental health.

	Intelligence quotient		SNAP-IV		CHQ		Family APGAR	
	B (95% CI)	p-value	B (95% CI)	p-value	B (95% CI)	p-value	B (95% CI)	p-value
Age (years)	0.79 (− 0.13, 1.71)	0.091	0.24 (− 0.72, 1.20)	0.621	0.03 (− 0.17, 0.11)	0.712	0.01(− 0.16, 0.18)	0.928
Sex (female vs. male)	0.16 (− 4.12, 4.44)	0.941	− 3.29 (− 7.26, 0.69)	0.104	0.00 (− 0.65, 0.65)	0.997	− 0.56 (− 1.35, 0.23)	0.161
Age of caregivers	0.24 (− 0.25, 0.73)	0.340	− 0.18 (− 0.64, 0.29)	0.462	− 0.04 (− 0.11, 0.04)	0.336	− 0.11 (− 0.20, − 0.02)	0.018
**Education of caregivers**								
High school or lower	Reference		Reference		Reference		Reference	
College	6.79 (2.04, 11.53)	0.005	− 0.44(− 4.81, 3.92)	0.842	− 0.79 (− 1.51, − 0.07)	0.033	1.36 (0.49, 2.24)	0.003
Master or above	10.90 (4.19, 17.60)	0.002	− 4.36 (− 10.55,1.83)	0.166	− 1.45 (− 2.46, − 0.44)	0.005	2.40 (1.17, 3.63)	0.000
**Allergic diseases**								
Allergic rhinitis	− 0.70 (− 7.24, 5.83)	0.832	− 1.22 (− 6.84, 4.40)	0.668	0.10(− 0.90, 1.10)	0.841	− 0.52 (− 1.74, 0.70)	0.399
Asthma	1.55 (− 4.86, 8.00)	0.634	1.66(− 3.89 7.21)	0.555	− 1.00 (− 1.99, − 0.00)	0.049	0.15 (− 1.06, 1.36)	0.807
Atopic dermatitis	− 0.12 (− 6.05, 5.81)	0.968	− 1.49 (− 6.64, 3.66)	0.569	− 0.18(− 1.09, − 0.74)	0.705	− 0.07 (− 1.18, 1.03)	0.897
Urticaria	− 7.48 (− 18.02, 3.06)	0.163	− 6.61(− 16.97, 3.76)	0.210	− 0.08 (− 1.67, 1.51)	0.923	− 0.58 (− 2.51, 1.35)	0.557

**Figure 1:**
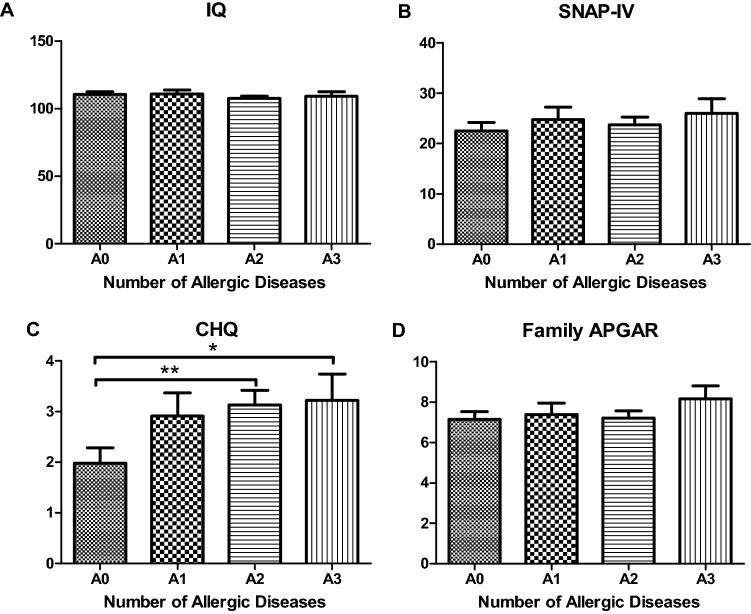
The relationship of numbers of atopic diseases and children’s development and caregivers’ mental health. A0: children without atopic disease, A1: child with one atopic disease, A2: children with two atopic diseases, A3: children with three or more atopic diseases.

Of the children with AD, 21.1%, 59.6%, and 19.3% of them had one atopic disease (A1), two atopic diseases (A2), and three or more atopic diseases (A3), respectively. The relationships between the number of atopic diseases and children’s development and caregivers’ mental health are shown in Fig. 1. Compared to caregivers of children without atopic disease (A0), the caregivers of the A2 (mean difference = 1.05, p = 0.005) and A3 groups (mean difference = 1.08, p = 0.045) had significantly higher CHQ scores. Except for CHQ, we observed no significant difference in cognitive scores, SNAP-IV, or family APGAR scores between the four groups.

## Discussion

This study is the first to investigate the potential effect of atopic diseases on cognitive development and parental stress. Inconsistent with our hypothesis, our data revealed that allergic rhinitis, asthma, atopic dermatitis, and urticaria were not associated with children’s cognitive profiles or ADHD symptoms. However, the caregivers of patients who had asthma experienced a greater mental health burden than those of patients without asthma. Furthermore, the numbers of atopic diseases had a dose–response effect on caregivers’ mental health.

Our data revealed that allergic rhinitis, asthma, atopic dermatitis, and urticaria were not associated with children’s cognitive profiles. While allergic rhinitis is a chronic disease that effects quality of life, we identified no negative effects on IQ^26^. Although asthma is a chronic disease and causes many respiratory problems, it also has no negative impact on IQ^14^. Nevertheless, certain neurocognitive symptoms are increased in children with moderate-to-severe atopic dermatitis, compared to healthy controls^22^. The relationships between urticaria and intelligence have not been well-established. Atopy is defined as a personal and/or familial tendency, usually in childhood or adolescence, to become sensitized and produce IgE antibodies in response to ordinary exposure to allergens, usually proteins. We consider that chronic atopic diseases may have detrimental effects on neurodevelopment. However, in line with previous studies, we have found that none of allergic rhinitis, asthma, atopic dermatitis, or urticaria have detrimental effects on children’s cognitive function.

In addition to cognitive function, we found that allergic rhinitis, asthma, atopic dermatitis, and urticaria were not associated with children’s ADHD symptoms. This finding contradicts current opinions raised by many researchers. A number of previous evidence has supported the association between ADHD and allergic/autoimmune diseases^10,11,27^. Various allergic diseases, like allergic rhinitis, atopic dermatitis, and asthma, have been associated with ADHD^16–19^. We have proposed some possible explanations for this discrepancy. Previous epidemiological studies primarily investigated the relationship between ADHD diagnosis and atopic diseases. This kind of reimbursement data may be influenced by a detection bias. For example, patients with atopic diseases who regularly followed up in the pediatrics outpatient-department (OPD) may be more likely referred to a child psychiatrist and be diagnosed as ADHD. In contrast, the ADHD symptoms in our study were presented as SNAP-IV score (continuous variable). Furthermore, the participants were consecutively recruited in OPD, and no significant difference in ADHD symptom severity was found between patients and controls.

This study is the first to provide evidence related to the profiles of parenting stress in the caregivers of children with atopic diseases. Our data revealed that caregivers of children who had asthma or multiple atopic diseases suffered from greater parenting stress than the caregivers of children who did not have any atopic diseases. Negative experiences with asthma care and the unpredictability of the disease outcomes impair the ability of caregivers to adapt successfully to their caregiving role and encourage perceptions that they cannot cope with this illness^28,29^. Moreover, individuals with multiple atopic diseases cope with a significant psychosocial burden, in addition to dealing with the medical aspects of the disease^24^. Caring for children with multiple atopic diseases can be a time-consuming task that can impair caregivers’ personal relationships, as well as decrease psychosocial functioning^23^. Deficient sleep with poor quality in the caregivers of children with chronic illnesses may have a significant impact on their health and well-being, as well as on their caregiving responsibilities^25^. Pursuant to our findings, healthcare providers should better target support to these caregivers of children with asthma or multiple atopic diseases so that they can better care for their children.

This study has certain limitations. First, this is a cross-sectional study, so the duration of illness and possible medications were not recorded. Whether the children’s outcomes and caregivers’ mental health were influenced by the treatment outcome is unknown. Second, atopic diseases were only recorded as a categorical variable (with or without), but the symptom severity on cognition or parenting stress was not assessed herein. Third, we did not record physical or neurodevelopmental comorbidities. Whether other comorbidities (i.e., developmental delay or epilepsy) may actually influence or moderate children’s cognitive development warrants further investigation. Fourth, the sample size was small (especially the control group), which reduced the statistical power of this study.

Allergic rhinitis, asthma, atopic dermatitis, and urticaria were not associated with children’s cognitive profiles or ADHD symptoms. This result is good news for caregivers and patients with atopic diseases, reassuring them that their atopic diseases will have no effect on their cognitive development or ADHD symptoms. However, the caregivers of patients who had asthma or multiple atopic diseases may feel stress about the physical or psychological burden of caring for their children. These caregivers may require support or help to overcome that stress.

## Methods

### Participants in a clinical setting

We recruited a total of 109 patients with atopic diseases from the Department of Pediatrics, Kaohsiung Chang Gung Memorial Hospital, Taiwan or communities near the hospital. Patients who had one or more atopic diseases (allergic rhinitis, asthma, atopic dermatitis, or urticaria) were recruited by a properly trained allergist clinician.

The 82 control subjects consisted of healthy children from the communities surrounding Kaohsiung Chang Gung Memorial Hospital or of children suffering from upper respiratory tract infection (URI) whose symptoms were currently in remission. We excluded any patients with atopic diseases (allergic rhinitis, asthma, atopic dermatitis, or urticaria) or other major physical illnesses (such as genetic, metabolic, or infectious conditions).

### Neurocognitive assessments

A developmental or cognitive assessment was administered to each patient and control subject by an experienced child psychologist in a room designed to reduce testing condition variables. Subjects under the age of 4 years old were assessed using the Mullen Scales of Early Learning (MSEL)^30^; subjects between the ages of 4 and 7 years old were examined using the Wechsler Preschool and Primary Scale of Intelligence-Fourth Edition (WPPSI-IV)^31^; and subjects older than 7 years old were tested using the Wechsler Intelligence Scale for Children-Fourth Edition (WISC-IV)^32^. The Early Learning Composite score of MSLE and the Full-Scale Intelligence Quotient (FSIQ) of WPPSI-IV and WISC-IV were considered the intelligence quotient score. The patients’ caregivers were requested to complete the following questionnaires which assess the ADHD symptoms severity and mental health burden.

*The Chinese Version of the Swanson, Nolan, and Pelham IV Scale (SNAP-IV)*, a 26-item questionnaire, is commonly used for evaluating ADHD symptoms and severity. The questionnaire can be completed by either parents or teachers^33^. The 26 items consist of nine items for inattention, nine items for hyperactivity and impulsivity and eight items that concern oppositional defiant disorder symptoms, as defined in the DSM-IV-TR. Each item is scored on a four-point Likert scale (from 0 to 3). The Chinese version of the SNAP-IV has been reported to have satisfactory reliability and concurrent validity^34^.

*The Chinese Health Questionnaire (CHQ-12)*, a 12-item self-report questionnaire, was modified from the General Health Questionnaire^35^. This instrument has been widely used to identify those who have minor psychiatric disorders in both primary care and community settings. This measure has been proven to have good reliability and validity^35^.

*Family APGAR*, a five-item measure often used to measure family well-being^36^, was completed by the primary caregiver in each of the five areas by using a three-point Likert scale, ranging from 0 (low satisfaction) to 2 (high satisfaction). The Mandarin version has adequate internal reliability and validity.

### Statistical analysis

All data processing and statistical analyses were performed using the Statistical Package for Social Science (SPSS) software, Version 21.0 (SPSS, Chicago, IL, USA). Two-tailed *p* values < 0.05 were considered statistically significant.

We adopted the Chi-square test to compare differences in categorical variables between patients with atopic diseases and healthy controls. We compared continuous variables between the two groups through an independent *t*-test. Multiple linear regression was carried out to observe the effect of atopic diseases on children’s cognitive function and caregivers’ mental health. The dependent factors were set as a cognitive score, total scores of SNAP-IV, CHQ, and family APGAR. The cognitive scores were set as the ELC scores of the MSEL or the FSIQ scores of the WPPSI or WISC-IV. The four atopic diseases (allergic rhinitis, asthma, atopic dermatitis, or urticaria) were considered the independent variables, and we controlled the confounding effects of children’s age and sex and caregivers’ age and education levels.

To investigate whether the number of atopic diseases had dose–response or dose-related effects on children’s and caregivers’ outcomes, we categorized the participants into children who had no atopic disease (A0), children who had one atopic disease (A1), children who had two atopic diseases (A2), and children who had three or more atopic diseases (A3). Furthermore, we used one-way ANOVA with an LSD post-hoc test to examine the difference in cognitive, SNAP-IV, CHQ, and family APGAR scores between the aforementioned groups.

### Ethical approval

This study was approved by the Chang Gung Memorial Hospital’s Internal Review Board (IRB No.201700509B0), and we obtained written informed consent from the parents or guardians of all participating children. All methods were performed in accordance with the relevant guidelines and regulations by the Declaration of Helsinki.
